# ^10^Be-inferred paleo-denudation rates imply that the mid-Miocene western central Andes eroded as slowly as today

**DOI:** 10.1038/s41598-018-20681-x

**Published:** 2018-02-02

**Authors:** Andrea Madella, Romain Delunel, Naki Akçar, Fritz Schlunegger, Marcus Christl

**Affiliations:** 1 Institute of Geological Sciences, University of Bern, Bern, CH-3012 Switzerland; 2 Laboratory for Ion Beam Physics, ETH Zurich, Zurich, CH-8049 Switzerland; 3 Present Address: Department of Geosciences, University of Tübingen, Tübingen, D-72074 Germany

## Abstract

Terrestrial cosmogenic nuclide concentrations of detrital minerals yield catchment-wide rates at which hillslopes erode. These estimates are commonly used to infer millennial scale denudation patterns and to identify the main controls on mass-balance and landscape evolution at orogenic scale. The same approach can be applied to minerals preserved in stratigraphic records of rivers, although extracting reliable paleo-denudation rates from Ma-old archives can be limited by the target nuclide’s half-life and by exposure to cosmic radiations after deposition. Slowly eroding landscapes, however, are characterized by the highest cosmogenic radionuclide concentrations; a condition that potentially allows pushing the method’s limits further back in time, provided that independent constraints on the geological evolution are available. Here, we report 13–10 million-year-old paleo-denudation rates from northernmost Chile, the oldest ^10^Be-inferred rates ever reported. We find that at 13–10 Ma the western Andean Altiplano has been eroding at 1–10 m/Ma, consistent with modern paces in the same setting, and it experienced a period with rates above 10 m/Ma at ~11 Ma. We suggest that the background tectono-geomorphic state of the western margin of the Altiplano has remained stable since the mid-Miocene, whereas intensified runoff since ~11 Ma might explain the transient increase in denudation.

## Introduction

Denudation plays a fundamental role in the central Andean topographic evolution, because it removes and redistributes mass^1^, thereby influencing the geodynamic evolution of the orogen^2,3^. However, while the long-term paleo-climate and the chronology of surface uplift have long been investigated and discussed^4,5^, direct quantitative observations of the past erosional history of the central Andes are lacking and cannot be inferred from climate data, because relationships between climate and denudation are non-linear^6^. Therefore, a better understanding of the tectono-geomorphic evolution of the orogeny depends on our ability to fill this information gap, and heavily relies on investigations of the past erosional products of the Andes.

Sand collected along river-beds constitutes a mixture of the rocks undergoing erosion in the source catchment. This is the case for both modern stream sediments and erosional products preserved in stratigraphic records. In these detrital sediments, the concentration of *in-situ* produced cosmogenic ^10^Be is therefore representative of the catchment spatial average^7,8^ and it is inversely proportional to the denudation rate at work on the hillslopes^9,10^. Accordingly, analyses of ^10^Be concentrations in fluvial sediments allow inferring spatially averaged catchment-wide denudation rates in active rivers^8,10–12^. The described approach can also be applied to fluvial sedimentary archives, provided that independent constraints allow corrections for nuclides’ production and decay following deposition^7,13–16^. Here, we anticipate that paleo-denudation rates obtained from sedimentary archives of the Andean piedmont would give unprecedented insights into the history of denudation and, hence, on the past tectono-geomorphic state of their source area. We target the Late Miocene El Diablo formation, found in northern Chile at ca. 18°–20°S (Fig. 2), in order to reconstruct the denudation history of the western margin of the Altiplano at this latitude.

**Figure 1:**
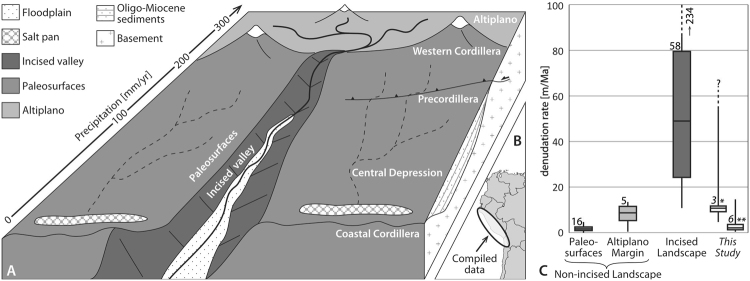
(**A**) Block diagram displaying the main tectono-geomorphic domains of the western escarpment of the central Andes at 15°–22°S. Altiplano: high-elevation, low-relief, non-incised. Western Cordillera: highest volcanic peaks, minimal incision. Precordillera: active W-verging thrust system, moderate incision. Central Depression: preserved non-incised paleosurfaces with ephemeral streams and endorheic drainage, decoupled from the deeply-incised valleys hosting perennial streams. Coastal Cordillera: actively uplifting relict erosional surface. (**B**) Spatial extent of the compiled data set of modern denudation rates^15,16,19,20^, for more details the reader is referred to the supplementary data. (**C**) Boxplot displaying the statistics of the compiled ^10^Be-inferred modern denudation rates from the different tectono-geomorphic domains and the mid-Miocene paleo-denudation rates from this study (single asterisk: samples 10.5–11.5 Ma-old; double asterisk: all other samples). The solid vertical lines indicate minimum and maximum values; the lower and upper end of each box indicates the first and the third quartile, respectively; the solid horizontal line indicates the median value; the number next to each box indicates the population. This figure was created using the software Adobe Illustrator^®^ CS5 (www.adobe.com/products/illustrator.html), licensed to the University of Bern.

**Figure 2:**
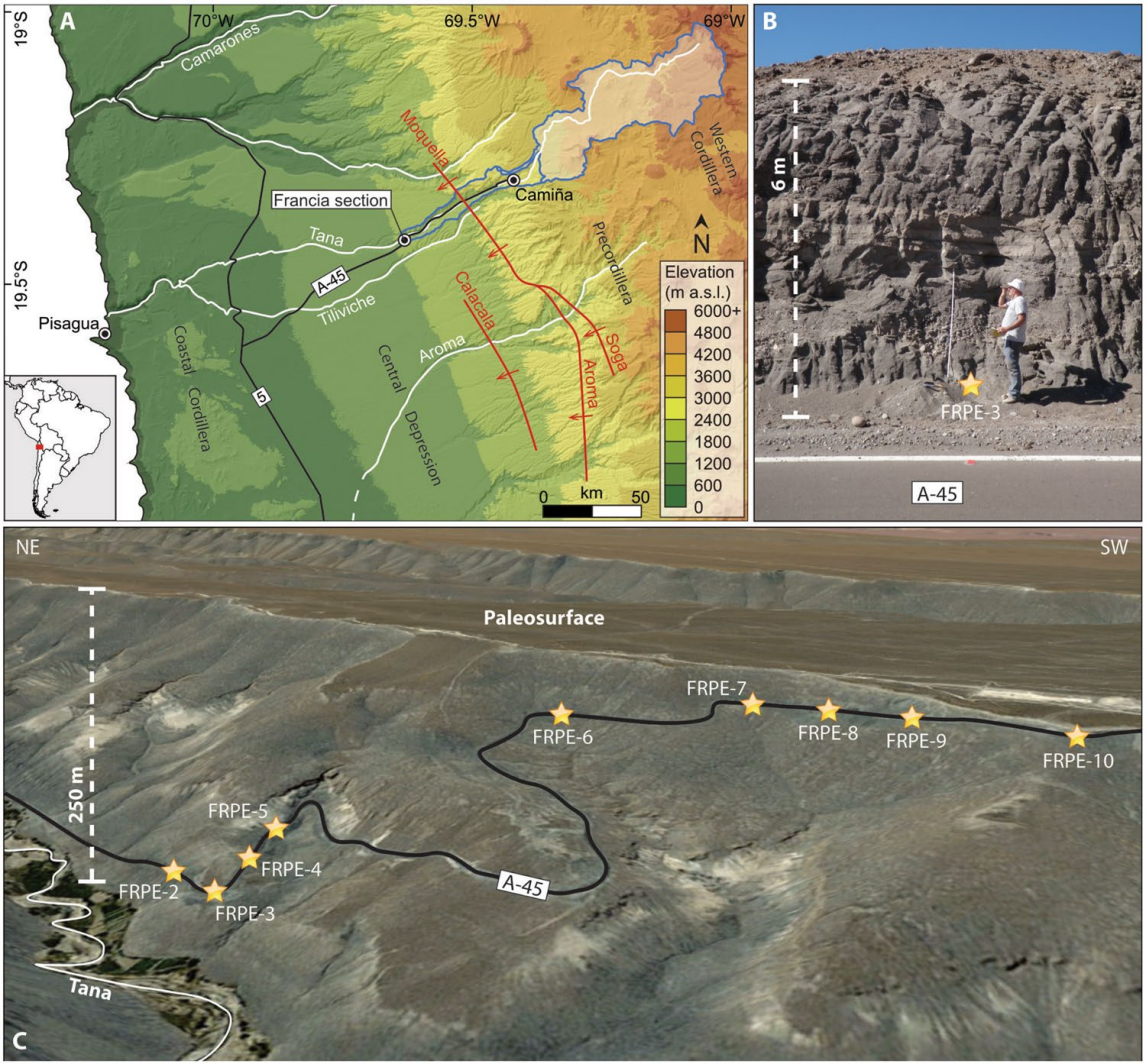
(**A**) Location of the sampled Francia section within the study area. The along-strike tectono-geomorphic domains are indicated (Coastal Cordillera, Central Depression, Precordillera, Western Cordillera). Main roads are displayed by black lines, white lines indicate rivers, red lines indicate the main WSW-vergent flexures^29^. The blue polygon outlines the current upstream catchment, with the white shaded area indicating elevations above 3000 m a.s.l. (**B**) Example of sampling spot at the bottom of the road cut. (**C**) View of the southern flank of the Tana Valley, where each sample location along the road is displayed. The depth of the incision between the top of the El Diablo fm and the modern valley bottom is indicated to the left. Image data: Google, CNES/Airbus and Landsat/Copernicus. This figure was created using the software ArcGIS 10.1 (www.arcgis.com) and Adobe Illustrator^®^ CS5 (www.adobe.com/products/illustrator.html), both licensed to the University of Bern.

The El Diablo formation (fm) is a fluvio-lacustrine suite deposited between ca. 17–10 Ma in the Central Depression^17^, the sedimentary basin situated between the Western Cordillera of the Andes and the Coastal Cordillera (Fig. 1). The El Diablo fm overlies with an unconformity a widespread Oligo-Miocene volcanoclastic sequence referred to as the Oxaya formation^17–20^. It exhibits substantial spatial thickness variations as well as changes in stratigraphic architecture, thickening from few tens of meters at 18°S to more than 200 m at 19°–20°S, and offering best exposure conditions along the Francia section^21,22^. The Francia section, located at 19.25°S along the road A-45 to Camiña (Fig. 2), is a type locality for the proximal facies of the upper El Diablo fm, where it displays an alternation of mudstones, sandstones and conglomerates sourced in the western margin of the Altiplano and deposited at roughly 13–10 Ma^17,21^. Despite the remarkably old depositional age of the analysed samples, the existing independent constraints on the history of burial and exhumation allow reconstructing a history of paleo-denudation that extends beyond previously reported time limits for ^10^Be^13,16^. Following the approach recently fostered by a number of authors^14,16,23–25^, reliable 13–10-Ma-old denudation rates of the western central Andes are calculated and compared to a compilation of present-day denudation rates from the same area. The modern pattern of denudation will serve as a benchmark to discuss the past geomorphic state of the analysed samples’ source area. Furthermore, we will explore whether the change towards a wetter climate in the Western Cordillera at ~11 Ma, recorded by a shift from ephemeral to perennial sedimentation in the El Diablo fm^22^, had a measurable impact on the denudation rates in the source area at that time.

## Results

### Geologic history of the region

The inversion of cosmogenic ^10^Be concentrations into paleo-denudation rates requires precise quantitative constraints on the geologic history of the study area after deposition of the sampled sediments. In this paragraph, we compile a synthesis of the known chronology of surface uplift and exhumation for the western central Andes between 19°–20°S.

Fluvial deposition of the El Diablo fm ceased at ~10 Ma. The ~10 Ma-old depositional surface (Aggradational Surface 4 in Evenstar *et al*.^26^) was subsequently preserved likely due to the hyperarid conditions^26^, and finally topped by the andesitic flow of the Lava de Tana at 10–8 Ma^17,27^ a few km north of the Francia section. At 7–5 Ma the Tana river started incising the Tana canyon from the eastern margin of the Central Depression headward, likely in response to tectonic tilting of the margin of the Western Cordillera^26,28–30^. This phase of fluvial down-cutting was limited by the presence of a paleo-lake located at the western rim of the Central Depression, which was setting a local base level for the upstream reach until its abandonment at ~3 Ma^31^. Overflow of the paleo-lake and related base level drop down to the Pacific Ocean initiated a second wave of incision in this reach, which likely strengthened thereafter and continued through Quaternary times^31^, thereby progressively exposing the El Diablo fm along the Francia section (Fig. 2).

Tectonic activity at the eastern edge of the Central Depression is expressed by a series of WSW-verging flexures^32^ that have accommodated a maximum of 3 km of shortening since the early Miocene^33^. Based on structural investigations, Farias *et al*.^29^ showed that up to 1900 m of uplift were accomplished since 25 Ma through shortening along the flexures Soga, Aroma and Calacala (Fig. 2). According to the same authors, relative uplift along these flexures has controlled the formation of accommodation space to the west, which has been filled syntectonically by the El Diablo fm^17^. The Moquella flexure intercepts the Tana Valley ~12 km east of the Francia section and represents the northward continuation of the Aroma flexure^29^ (Fig. 2). Miocene uplift was therefore most likely focused east of the Moquella flexure, whereas little to zero vertical surface uplift has affected the Francia section since ~13 Ma, as suggested by cosmogenic noble gas concentrations in boulders west of the Calacala flexure^34^.

The present-day Tana canyon is characterized by a ca. 100-m-wide valley flat, bordered by steep and densely gullied valley flanks that undergo a spatially-averaged denudation rate of 32 ± 6 m/Ma, as revealed by *in-situ* ^10^Be concentration in detrital material^35^. Farther upstream, the deeply dissected Tana valley is separated from the non-dissected headwater reach by a distinct knickzone. Compared to the other major fluvial canyons to the north, the Tana valley exhibits no major gravitational collapse and only minimal vertical incision^26^, suggesting that mass-wasting processes do not constitute the main erosional mechanism in the study area.

### Paleo-denudation rates

Inherited ^10^Be accumulated within surface materials during exposure in the paleo-catchment should represent >50% of the total measured concentration in order to yield accurate paleo-denudation rate estimates^36^. This implies that sediments originating from slowly eroding catchments, quickly buried and only marginally re-exposed, bear a high potential to yield ^10^Be-inferred paleo-denudation rates even several million years after deposition. We collected 9 samples from the fluvial sandstones of the El Diablo formation exposed along the Francia section^21,22^ (Fig. 2). The cosmogenic ^10^Be concentrations extracted from these samples were corrected for post-depositional contributions and used to infer mid-Miocene paleo-denudation rates. For each of the collected samples, we assume a path that involves (i) exposure and denudation on the hillslopes, followed by (ii) transport and deposition at a known time, (iii) progressive burial and (iv) progressive re-exhumation and exposure to cosmic rays during the incision of the Tana canyon. The constraints on the parameters that govern the outlined steps are discussed in the methods section, and the sensitivity of the paleo-denudation estimates to possible variations in some of these parameters is reported in the supplementary information.

**Figure 3:**
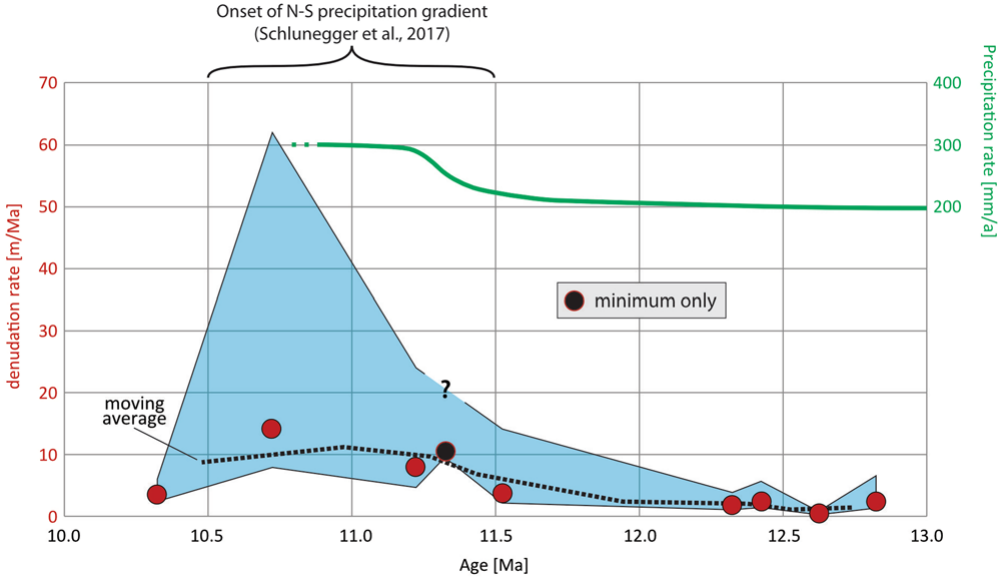
^10^Be-inferred paleo-denudation rates of the Western Cordillera between 13–10 Ma. The light blue shaded area shows the 1σ solution space. The black dashed line displays the 2-samples moving average. The precipitation rates reconstructions inferred by Schlunegger *et al*.^22^ are plotted in green on the right vertical axis. This figure was drawn using the software Adobe Illustrator^®^ CS5 (www.adobe.com/products/illustrator.html), licensed to the University of Bern.

The measured ^10^Be concentrations (*C*_*measured*_) range between 2 × 10^4^ and 5 × 10^4^ at/g with relative uncertainties of 4–30% (Table 1). The post-depositional signal makes up less than 48% of the fossil concentration in all samples, except for FRPE-8 and -9, where the post-depositional signal makes up ~70%, and FRPE-7, where the post-depositional concentration is 86% of *C*_*measured*_ (Table 2). Our estimates indicate that these post-depositional concentrations are mostly due to the samples’ exhumation (*C*_*incision*_), whereas concentrations due to exposure to cosmic rays between deposition and complete burial (*C*_*burial*_) represent less than 2% of the measured concentrations (Table 2). This allows extracting rather accurate estimates of paleo-denudation rates for 6 out of 9 samples, whereas for FRPE-8 and -9 positive relative uncertainties exceed 200%. Results for sample FRPE-7 are only meaningful as a minimum estimate. Despite these rather large uncertainties, the paleo-denudation rates inferred for the time between 13–10 Ma cluster in two groups with different orders of magnitude, exhibiting the highest values in ~11 Ma-old material (Fig. 3). The samples dated to 12.8–11.4 Ma (FRPE-2 to -6) yield paleo-denudation rates ranging between 0.3–14.1 m/Ma, with a median value of 2.4 m/Ma (Table 2, Fig. 1). Samples deposited at 11.4–10.5 Ma (FRPE-7, -8 and -9) yield rates ranging between 4.7–62.0 m/Ma, with a median value of 11.5 m/Ma. The paleo-denudation rate estimated for the youngest sample (FRPE-10, 10.3 Ma-old) falls in the same range as the oldest ones.

**Table 1:** Information on processed samples, measured ^10^Be/^9^Be ratios and ^10^Be concentrations.

Sample	Dissolved qtz [g]	^9^Be spike [mg]	^10^Be/^9^Be [10^−14^]			^10^Be *C*_*measured*_ [10^4^ at/g]		
FRPE-2	9.0624	0.1741	1.75	±	0.38	1.96	±	0.49
FRPE-3	14.6787	0.1746	7.00	±	1.11	5.38	±	0.88
FRPE-4	13.5546	0.1745	2.55	±	0.60	2.00	±	0.52
FRPE-5	7.2132	0.1731	1.86	±	0.44	2.62	±	0.70
FRPE-6	7.1267	0.1741	1.89	±	0.49	2.72	±	0.80
FRPE-7	7.4761	0.1749	1.49	±	0.29	1.98	±	0.46
FRPE-8	11.6216	0.1724	2.81	±	0.27	2.56	±	0.27
FRPE-9	14.0992	0.1749	2.71	±	0.26	2.06	±	0.22
FRPE-10	37.1068	0.1699	16.42	±	0.65	4.95	±	0.20

The concentration of the used ^9^Be spike is 1 g/l. Displayed quantities for *C*_*measured*_ have been corrected for the long-term average ^10^Be/^9^Be blank ratio of 2.3 × 10^−15^ ± 0.1 × 10^−15^ measured for the clean laboratory where sample preparation took place (Institute of Geological Sciences, University of Bern).

**Table 2:** Results of the calculations of best fit paleo-denudation rates.

Sample	Age [Ma]	*C*_*measured*_ [10^4^ at/g]	*C*_*burial*_ [10^2^ at/g]_*s*_		*C*_*incision*_ [10^4^ at/g]		*C*_*hillslope*_ [10^6^ at/g]	Paleoden. rate *ε*_*p*_ [m/Ma]		
				% *C*_*meas*_		% *C*_*meas*_			−1σ	+1σ
FRPE-2	12.80 ± 0.03	1.96 ± 0.49	1.54 ± 0.54	1%	0.84 ± 0.18	43%	8.01 ± 4.91	2.3	1.0	4.2
FRPE-3	12.60 ± 0.13	5.38 ± 0.88	1.71 ± 0.61	0%	0.80 ± 0.18	15%	29.82 ± 12.13	0.5	0.2	0.4
FRPE-4	12.40 ± 0.07	2.00 ± 0.52	1.89 ± 0.65	1%	0.64 ± 0.15	32%	7.89 ± 4.29	2.4	1.0	3.2
FRPE-5	12.30 ± 0.07	2.62 ± 0.70	1.99 ± 0.68	1%	0.71 ± 0.15	27%	10.59 ± 5.52	1.7	0.6	2.2
FRPE-6	11.50 ± 0.13	2.72 ± 0.80	3.18 ± 1.03	1%	1.28 ± 0.27	47%	5.22 ± 3.71	3.8	1.6	10.4
**FRPE-7**	11.30 ± 0.13	1.98 ± 0.46	3.59 ± 1.15	2%	1.67 ± 0.33	84%	0.96 ± 2.00	**10.5**	**1.0**	**1.0**
FRPE-8	11.20 ± 0.13	2.56 ± 0.27	3.80 ± 1.21	1%	1.71 ± 0.35	67%	2.61 ± 1.70	8.0	3.3	16.0
FRPE-9	10.70 ± 0.36	2.06 ± 0.22	5.01 ± 1.75	2%	1.42 ± 0.30	69%	1.50 ± 1.14	14.2	6.3	47.8
FRPE-10	10.30 ± 0.36	4.95 ± 0.20	6.22 ± 2.11	1%	2.12 ± 0.41	43%	5.61 ± 2.22	3.5	1.1	2.5

The estimated post-depositional concentrations are also displayed as fractions of the total measured concentrations. Bold values indicate only minimum estimates.

### Compilation of present-day denudation rates

Compilation of ^10^Be-inferred erosion rates (Table S1) shows that the incised catchments sourced in the Western Cordillera and western margin of the Altiplano between 5°–20°S currently erode at several tens to hundreds of meters per Ma (Fig. 1). Conversely, bedrock and detrital samples collected on the non-incised Altiplano at 5°, 14° and 18°S yield erosion rates ranging between 1–12 m/Ma (Fig. 1). Moreover, in the large non-incised areas decoupled from the deeply-incised canyons, bedrock and boulder surfaces undergo erosion at rates mostly lower than 1 m/Ma, which is two orders of magnitude lower than in the deeply-incised valleys (Fig. 1).

## Discussion

Precipitation increase is generally expected to result in intensified surface erosion, as well as higher rates of tectonic activity and seismicity are considered prone to exert a positive forcing on denudation rates^37,38^. However, linking ^10^Be-inferred spatial patterns of denudation to the landscape’s climatic and/or tectonic state has been proven a complex task^39^, because these millennial scale erosion rates might only offer a snapshot of the transient geomorphic response to a longer-lasting forcing^6^. The data compilation reported here, however, evidences that present-day denudation in the steep fluvial canyons occurs at substantially higher rates compared to the non-incised surfaces of the Altiplano. Here, consistently low denudation rates seem to be unrelated to precipitation changes (Fig. 1), but likely linked to the shallow slope angles and to the location upstream of the knickzone^40^. Extremely low denudation rates on the non-incised paleosurfaces of the Central Depression (Fig. 1), moreover, similarly correlate with low relief. The compiled data therefore allows quantitatively discriminating between (i) incised landscapes, undergoing erosion at 20–100 m/Ma, and (ii) non-incised landscapes, undergoing erosion at 0–10 m/Ma (Fig. 1). Our paleo-denudation estimates suggest that the fluvial deposits of the Francia section, spanning 13–10 Ma and sourced in the Western Cordillera and the western margin of the Altiplano^21,22^, have been derived from a paleo-catchment characterized by denudation rates mostly in the order of 1–10 m/Ma (Table 2; Figs 1 and 3). We therefore propose that such low denudation rates for the depositional time of the upper El Diablo fm are best explained through the presence of a long-term stable and non-incised landscape at the western margin of the Altiplano, and that this landscape form has been maintained for at least ~13 Ma. These interpretations are in agreement with a scenario in which the present-day crustal thickness and elevation of the western Altiplano had been reached by mid-Miocene times^41^, after which the tectono-geomorphic state did not significantly change until today.

Inferring paleo-runoff from the paleo-denudation rates would be speculative, because erosion of the present-day Altiplano margin has been shown not to correlate with annual precipitation^40^. Nevertheless, based on a shift in depositional style of the El Diablo fm from debris-flow matrix-supported conglomerates to clast-supported braided river conglomerates, Schlunegger *et al*.^22^ inferred a change from ephemeral to perennial fluvial runoff north of ~20° S at ca. 11 Ma. The same authors attributed this change to intensified orographic precipitation due to the growth of the Andes, as indicated by atmospheric circulation models^42^. Although the data from 11.5–10.5 Ma-old samples bear large uncertainties, they reveal a coeval transient increase of denudation rates exceeding 10 m/Ma (Fig. 3). This pattern seems to be further corroborated by the sensitivity tests presented as supplementary material. Here, we discard that intensified volcanic activity, also occurring at ~11 Ma in the Altiplano-Puna Volcanic Complex^4^, might have perturbed the denudation rates record. This choice is justified because one would expect a larger relative abundance of lahar interbeds in the post-11-Ma-old deposits, supplied by debris flow processes sourced on the volcanoes. This is not consistent with observations as the shift towards faster upstream denudation was associated with a change from debris flow to fluvial processes (and not vice versa). We likewise discard a possible enlargement of the paleo-catchment through stream cannibalization and headward retreat as alternative driver for larger discharge and faster denudation at 11 Ma, because the observed shift from debris flow to fluvial processes, which is associated to larger relative runoff volumes^43^, occurred over a broader scale across the western central Andes^22^. Conversely, if stream cannibalization and incorporation of steeper areas into the drainage basin had amplified erosion, the related shift in sedimentary fabric would have been a localized and strongly time-transgressive feature, as outlined by Schlunegger *et al*.^22^. We are thus left with a scenario where the increase in precipitation rates from 200 to 300 mm/a at ~11 Ma^22^ (Fig. 3) initiated an erosional pulse in the source area from ~2 m/Ma to >10 m/Ma, as recorded by the ^10^Be dataset. After 10.5 Ma, the observed decrease in paleo-denudation rates (Fig. 3) does not require a decrease in annual runoff in the source area, because the discussed intensification of denudation at ~11 Ma may simply represent a transient response to the climate shift. Numerical predictions by Tucker and Slingerland^44^ have shown that a shift to wetter climate induces an upstream expansion of the channel network within the drainage basin, resulting in a short-term erosional transient.

We conclude that denudation in the landscape upstream of the Francia section remained in the same order of magnitude (<10 m/Ma) over millions of years, and propose that precipitation increase at ~11 Ma^22^ might have caused a short-term pulse of erosion, which did not significantly perturb the long term pattern of very low denudation rates at the western margin of the Altiplano. This study also suggests that the presence of a stable low-relief non-incised landscape at the western margin of the Altiplano can be traced back to the mid-Miocene. These results represent the oldest ^10^Be-inferred paleo-denudation rates reported so far, and indicate that terrestrial cosmogenic nuclides measured in well-studied geological archives are a promising tool to gain insights into the past long-term geomorphic state of orogens.

## Methods

### Paleo-denudation rates

We collected 9 rock samples from the fluvial sandstones of the El Diablo formation, exposed in the Francia section of the Tana Valley^21,22^. Terrestrial cosmogenic ^10^Be concentrations in quartz extracted from these samples were corrected for post-depositional contributions and used to infer mid-Miocene paleo-denudation rates. The samples were prepared using the procedures described by Akçar *et al*.^45^ and references therein at the University of Bern. ^10^Be/^9^Be ratios were measured through accelerator mass spectrometry at the ETH Zurich^46^ and normalized to the ETH in-house standard S2007N^47^. After blank correction (Table 1), each measured concentration was corrected for post-depositional production, in order to isolate only the concentration inherited from denudation on the paleo-catchment’s hillslopes.

For each of the collected samples, we consider a path that involves (i) denudation on the hillslopes at the paleo-denudation rate *ε*_*p*_, which we aim to infer, followed by (ii) transport and deposition at time *t*_*d*_, (iii) constant progressive burial at a rate *A*_*r*_ and (iv) progressive re-exhumation at a rate *ε*_*i*_ during the incision of the Tana canyon since the time *t*_*i*_. In this scenario, the concentration *C*_*hillslope*_ inherited from paleo-denudation is described by^36^

1 $${C}_{hillslope}=\sum _{i,j,k}[\frac{{P}_{i,j,k}}{\lambda +{\rho }_{h}{\varepsilon }_{p}/{\Lambda }_{i,j,k}}]\,$$

where *i, j, k* represent each of the ^10^Be production paths (neutron spallation and both muons reactions), *ρ*_*h*_ is the density of the source rocks, and *Λ* is the characteristic attenuation length for the paths *i, j, k*. Moreover, *C*_*hillslope*_ has undergone exponential decay since deposition, and its relationship with the blank-corrected measured ^10^Be concentration *C*_*measured*_ is described by the following equation^36^:

2 $${C}_{hillslope}={C}_{hillslope{\rm{\_}}fossil}\cdot {e}^{{\lambda t}_{d}}=({C}_{measured}-{C}_{postdep})\cdot {e}^{{\lambda t}_{d}}$$

where *C*_*hillslope_fossil*_ represents the portion of *C*_*hillslope*_ remaining after radioactive decay, *C*_*postdep*_ is the portion of *C*_*measured*_ that equals the sum of all concentrations produced through exposure to cosmic radiations after deposition, and λ is the decay constant of ^10^Be. In the scenario described above, *C*_*postdep*_ is the sum of *C*_*burial*_ and *C*_*incision*_, the concentrations produced during burial and during canyon incision respectively. Given the ^10^Be production rate *P*, scaled to the burial location’s altitude and latitude, and the density of the unconsolidated sediments *ρ*_*b*_, *C*_*burial*_ can be calculated with the equation^14^

3 $${C}_{burial}=\sum _{i,j,k}[\frac{{P}_{i,j,k}}{\lambda -{\rho }_{b}{A}_{r}/{{\rm{\Lambda }}}_{i,j,k}}({e}^{-{t}_{d}\frac{{\rho }_{b}{A}_{r}}{{{\rm{\Lambda }}}_{i,j,k}}}-{e}^{-\lambda {t}_{d}})]$$

Using equation (3) is appropriate in case of deep burial beyond nuclide production. In this study, the sampled material was initially shallowly deposited in an aggrading system, but the use of equation (3), in opposition to the shallow-deposition equation described by Val and Hoke^36^, is justifiable because the related concentrations are negligible after several Ma of exponential decay.

In a rock body of density *ρ*_*i*_ subject to an erosion rate *ε*_*i*_ since the time *t*_*i*_, the ^10^Be concentration *C*_*incision*_ found at the depth *z* is described by^13^

4 $${C}_{incision}=\sum _{i,j,k}[\frac{{P}_{i,j,k}\,S}{\lambda +{\rho }_{i}{\varepsilon }_{i}/{{\rm{\Lambda }}}_{i,j,k}}{e}^{-{\rho }_{i}z/{{\rm{\Lambda }}}_{i,j,k}}\cdot (1-{e}^{-{t}_{i}(\lambda +{\rho }_{i}{\varepsilon }_{i}/{{\rm{\Lambda }}}_{i,j,k})})]$$

where *S* is the topographic shielding.

In order to solve equations (3) and (4) and lastly infer the paleo-denudation rate *ε*_*p*_, a number of variables need to be constrained. In the following paragraphs, we therefore discuss the constraints on *t*_*d*_, *A*_*r*_, *ε*_*i*_, *t*_*i*_ and other minor variables.

### Constraints on the history of deposition and burial

Schlunegger *et al*.^22^ have reassessed the chronology of the Francia section originally dated through magnetostratigraphy by von Rotz *et al*.^21^. Accordingly, the proximal fluvial sediments of the El Diablo fm outcropping in this location are considered to have been deposited at approximately 13–10 Ma. The 9 samples used in this study have been extracted from layers of known thickness and magnetic polarity (Fig. S8), for which we have determined a best-fit correlation to the Gradstein *et al*.^48^ reference chart within the software Cupydon^49^. The refined depositional ages (*t*_*d*_) of the collected samples are indicated in Table 3. For the calculation of *C*_*burial*_ we use an accumulation rate *A*_*r*_ of 70 m/Ma, the average throughout the whole section, whereas the density of the overburden *ρ*_*b*_ is assumed equal to 2.5 ± 0.1 g/cm^3^. The location of the burial sites, which are relevant for scaling the production rate (see below), have been assumed equal to the modern ones, and the elevations have been assumed to equal 95% of the modern ones with a relative uncertainty of 5%, in order to account for possible small amounts of uplift in the Central Depression since deposition (see above^29,34^).

**Table 3:** List of constraints used in the calculations described above.

Sample	Latitude	Longitude	Elevation	Depth	Age	Paleocatchment mean elevation	Burial rate	Burial site elevation	Time of incision	Local inc. rate	Shielding	Rock density	Sediment density
	°S	°W	m a.s.l.	*z* m	Ma	m a.s.l.	*A*_*r*_ m/Ma	m a.s.l.	*t*_*i*_ Ma	*ε*_*i*_ m/Ma	*S*	*ρ*_*h*_, *ρ*_*i*_ g/cm^3^	*ρ*_*b*_ g/cm^3^
FRPE-2	19.41364	69.62173	1671 ± 10	4.2	12.80 ± 0.03	3677 ± 350	70	1587 ± 84	5.9 ± 0.5	47 ± 4	0.7757	2.65	2.5 ± 0.1
FRPE-3	19.41399	69.62272	1678 ± 10	4.7	12.60 ± 0.13			1594 ± 84		46 ± 4	0.7491		
FRPE-4	19.41472	69.62165	1680 ± 10	7.0	12.40 ± 0.07			1596 ± 84		46 ± 4	0.7019		
FRPE-5	19.41542	69.6209	1684 ± 10	4.2	12.30 ± 0.07			1600 ± 84		45 ± 4	0.6279		
FRPE-6	19.41615	69.62157	1787 ± 10	4.7	11.50 ± 0.13			1698 ± 89		27 ± 2	0.7596		
FRPE-7	19.42436	69.62173	1823 ± 10	3.5	11.30 ± 0.13			1732 ± 91		21 ± 2	0.7341		
FRPE-8	19.42509	69.62328	1834 ± 10	4.5	11.20 ± 0.13			1742 ± 92		19 ± 2	0.7651		
FRPE-9	19.42571	69.6252	1850 ± 10	6.9	10.70 ± 0.36			1758 ± 93		17 ± 1	0.6844		
FRPE-10	19.42598	69.62815	1874 ± 10	4.7	10.30 ± 0.36			1780 ± 94		12 ± 1	0.7052		

We remark that accumulation is here assumed to be constant. Although additional re-exposure might have occurred between the deposition of a sample and of the following sedimentary layer, we show in the supplementary material (Fig. S1) that the possible effect of this component is negligible in this study.

### Constraints on the history of canyon incision

A number of studies concur that the Tana Valley has been incised since the time *t*_*i*_, comprised between 7–5 Ma^26,28,30^. Accordingly, we bracket the start of incision between the deposition of the 6.4-Ma-old strath terrace in the lower reach of the Tana canyon^50^, and the 5.4-Ma-old Carcote Ignimbrite, which filled incipient canyons of the Precordillera 150 km to the south^50^. The time of incision *t*_*i*_ used in our calculations therefore equals 5.9 ± 0.5 Ma. In order to estimate the variable *ε*_*i*_ in equation (4), we divide the elevation difference between the current top of the Francia section and the sample by the time of incision *t*_*i*_. This operation yields a time-averaged local incision rate for each sample, which represents how fast material has been removed from the rock column overlying each sampling location. The obtained local incision rates are indicated in Table 3. In the supplementary material, we additionally test the sensitivity of the paleo-denudation estimates with respect to two end-member scenarios that account for variable incision rates (Figs S2, S3). This test shows that independently from the incision history, the trend reported in Fig. 3 remains unaltered. The density of the rock *ρ*_*i*_ during canyon incision is assumed equal to 2.65 g/cm^3^. In addition, all the samples have been taken at the lowermost position of a 5 to 10-m-deep road cut, in order to minimize recent exposure. This depth *z* has been measured in the field, as well as the topographic shielding *S*. The samples’ coordinates and elevations during canyon incision are assumed to equal the present-day ones.

### Computation of the paleo-denudation rates

The best-fit paleo-denudation rates according to the described set of equations were calculated through a Monte Carlo simulation-based MATLAB package developed by Val and Hoke^36^, using the input parameters listed in Table 3. The script has been set to use its default sea-level high-altitude ^10^Be production rate of 4.09 ± 0.35 at/g/a^51^, scaled after Lifton *et al*.^52^. Muogenic production was set to follow the single exponential approximation proposed by Braucher *et al*.^53^. The Monte Carlo population was set to 10^5^. In this computation, the mean elevation of the paleo-catchment is a relevant parameter in order to scale the ^10^Be production rate at the time. Sensitivity tests performed by Val *et al*.^16^ reveal that if the paleo-catchment’s hypsometry is assumed similar to the present-day one, inferred paleo-denudation rates will exhibit variations within 30% from the actual rates. Such variations are within the range of the uncertainties obtained through our calculations (see results). Nevertheless, sedimentological reconstructions^17^ suggest that the hypsometry of the Tana paleo-catchment may have been remarkably different from the modern one, due to the absence of the incised canyon. We therefore utilize the mean elevation of a catchment similar to the present-day one (extracted from the 30-m-resolution ASTER GDEM digital elevation model^54^) but excluding elevations lower than 3000 m a.s.l. (Fig. 1). The resulting mean paleo-catchment elevation would equal 4027 m a.s.l. In addition, we account for the different possible scenarios of Andean uplift since 13 Ma^5^, thereby considering a minimum of zero and a maximum of 700 m of surface uplift. This maximum value has been chosen in accordance with estimates of post-13 Ma uplift in the Aroma region^29^ (Fig. 1). In summary, the used mean paleo-catchment elevation is 3677 ± 350 m a.s.l. as reported in Table 3.

### Other possible sources of uncertainty

The reported paleo-denudation estimates are obtained ignoring possible production at depth between the end of El Diablo deposition (~10 Ma) and the start of canyon incision (7–5 Ma). As we show in the supplementary material, this choice is justified by the fact that, even though ^10^Be production might have lasted for over ~3 Ma, the relative contribution after >6 Ma of radioactive decay would be negligible (Fig. S5).

It is known that spatial and temporal fluctuations of the magnetic field strength affect cosmogenic nuclide production rates^52^, although such variations are not reported for times preceding ~2 Ma. Assuming that the mid-Miocene magnetic field was subject to similar fluctuations, our calculation should take into account the uncertainty deriving therefrom. However, as shown in the supplementary data, slowly eroding landscapes are less sensitive to such variations, because short-term (1 ka) fluctuations are averaged over longer exposure times (>100 ka) (Figs S6, S7).

### Compilation of present-day denudation rates

We have compiled and recalculated ^10^Be-inferred current erosion rates for the western central Andes (Table S1). The published ^10^Be concentrations and the related topographic data^35,40,55,56^ have been used to re-estimate the current erosion rates through the online calculator formerly known as the CRONUS-Earth online calculator. The rates inferred for samples collected within streams sourced in the Coastal Cordillera have not been included in the compilation, due to the anomalously-low annual precipitation^57^. The rates inferred for bedrock samples from the Lauca-Perez ignimbrite have also been discarded, based on the anomalously-high erodibility of this lithology^20^.

## Electronic supplementary material

Supplementary Information
