# Supplementary material for: 10Be-inferred paleo-denudation rates imply that the mid-Miocene western central Andes eroded as slowly as today
Source: Sci Rep. 2018 Feb 2;8:2299. doi: 10.1038/s41598-018-20681-x (PMC5797110; doi:10.1038/s41598-018-20681-x)
Supplement: Supplementary file 1 — Supplementary Information [file 41598_2018_20681_MOESM1_ESM.pdf]

^{10}Be-inferred paleodenudation rates imply that the mid-Miocene western central Andes eroded as slowly as today

Andrea Madella[1,*], Romain Delunel[1], Naki Akçar[1], Fritz Schlunegger[1], Marcus Christl[2]

[1] Institute of Geological Sciences, University of Bern, Bern, CH-3012, Switzerland
[2] Laboratory for Ion Beam Physics, ETH Zurich, Zurich, CH-8049, Switzerland
*corresponding author (andrea.madella@geo.unibe.ch); current affiliation: University of Tübingen, Department of Geosciences, D-72074 Tübingen, Germany.

SUPPLEMENTARY DATA

Possible sources of uncertainty: sensitivity tests

As a complement for the results reported in the main text, the following paragraphs present additional model outputs that show the sensitivity to possible sources of uncertainty that are not taken into account in the preferred scenario.

Pre-burial exposure

The paleo-denudation rates reported in the main text are obtained assuming that burial starts when the sediment is deposited. However, it is possible that samples remain exposed for a certain amount of time before the next sedimentary layer arrives. Here, 50 ka of pre-burial ^{10}Be production are assumed, which after radioactive decay only amount to a maximum of ~1000 at/g to be removed from $C_{measured}$ before inferring $C_{inherited}$. The effect of this additional post-depositional component is shown in Fig.S1.

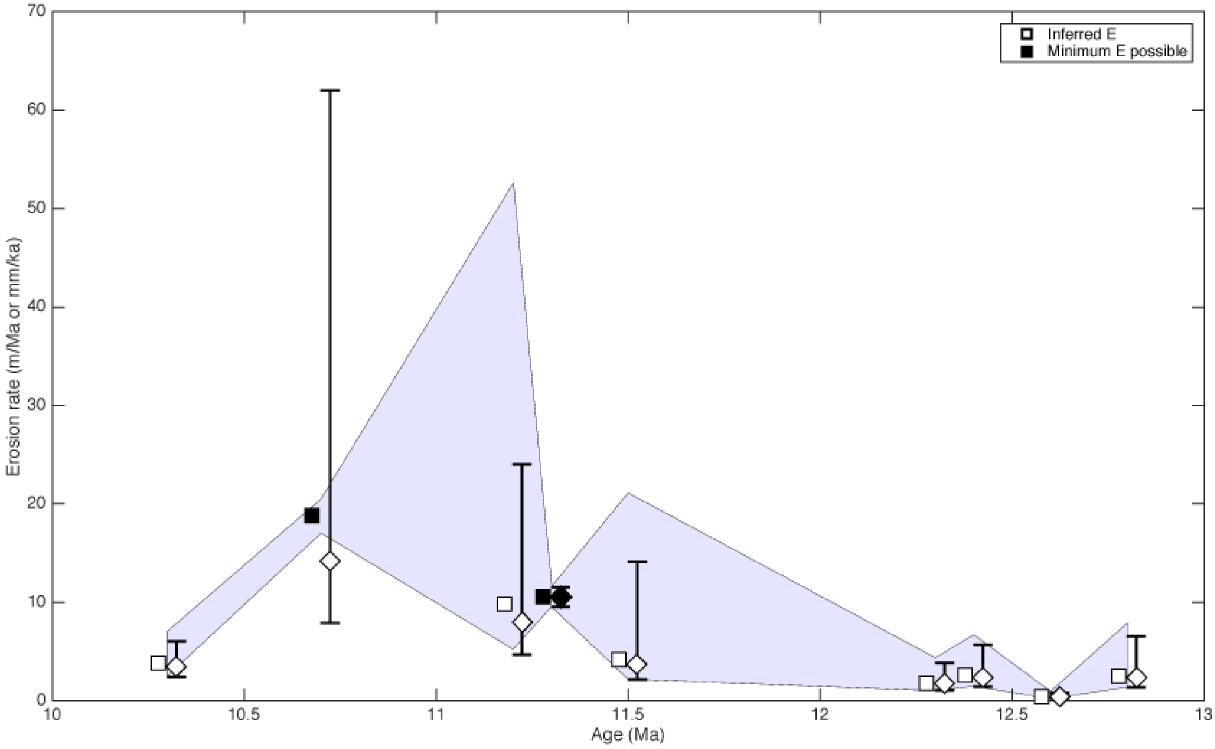

Figure S1. Paleo-denudation rates reported in the main text (diamonds with error bars), compared to a scenario where each sample was exposed 50 ka after deposition and before burial commenced. The resulting paleo-denudation rates (squares within blue 1σ envelope) show only minimal variations that do not affect the conclusions of this paper.

Variable canyon incision rate

The paleo-denudation rates reported in the main text are obtained assuming a constant incision rate during the phase of progressive exhumation, which equals the maximum sample depth *z* below the section's top (1947 m asl), divided by the incision time (5.9 ± 0.5 Ma). Here we show the possible effect of two consecutive 3-Ma-long incision phases at different rates, keeping the total cumulative incision equal to *z*. Fig. S2 relates to a scenario where the first phase carves 33% of *z* and the second one completes the remaining 66% (twice as fast). This scenario reduces the $C_{incision}$ component of the uppermost samples, hence increasing $C_{inherited}$ and reducing the paleo-denudation rates. Fig. S3 relates to the opposed scenario, where the first phase carves 66% of *z* and the second one completes the remaining 33%. In this case $C_{incision}$ becomes more relevant, and the youngest samples only yield minimum paleo-denudation rates.
Both scenarios show that the youngest and uppermost samples are the most sensitive to incision rate variations, evidencing that the related paleo-denudation rates can hardly be constrained. Nevertheless, the resulting trend remains analogous to the one reported in the main text, with an intensification of denudation rates above 10 m/Ma at ~11 Ma.

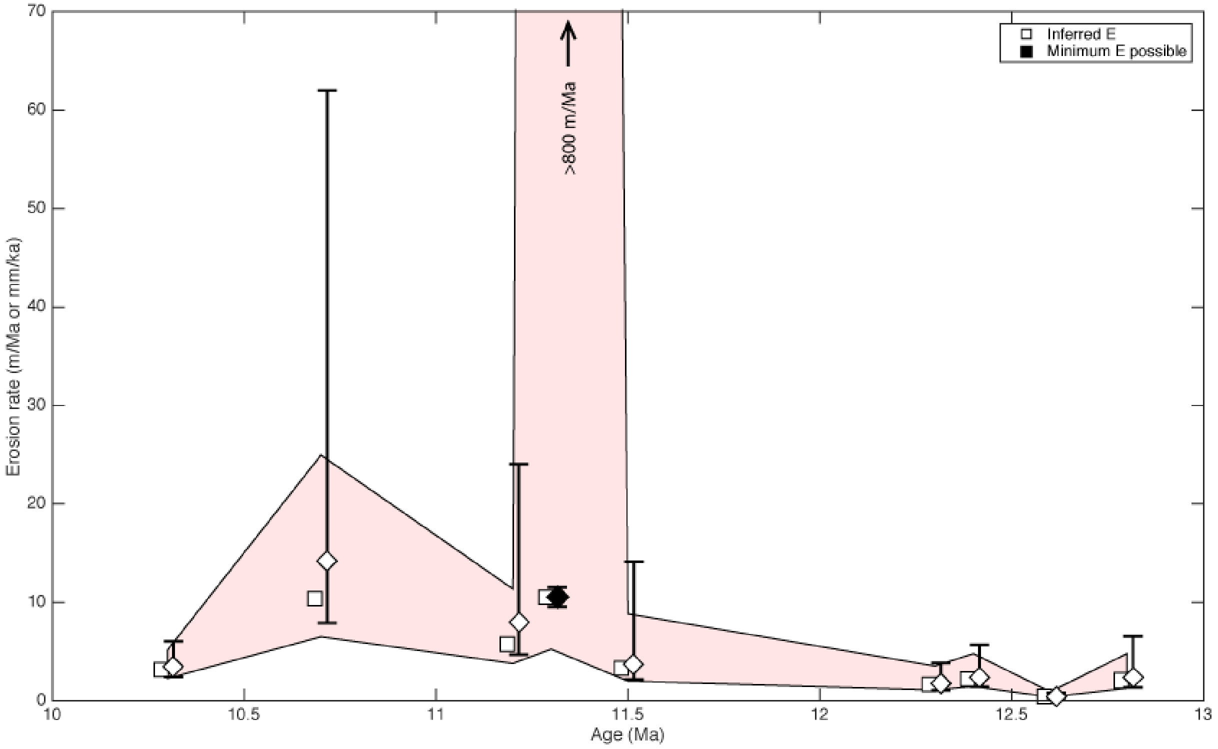

Figure S2. Paleo-denudation rates reported in the main text (diamonds with errorbars) compared to those obtained considering two 3-Ma-phases of canyon incision, where the second phase occurs at twice the rate (squares within rose 1σ envelope).

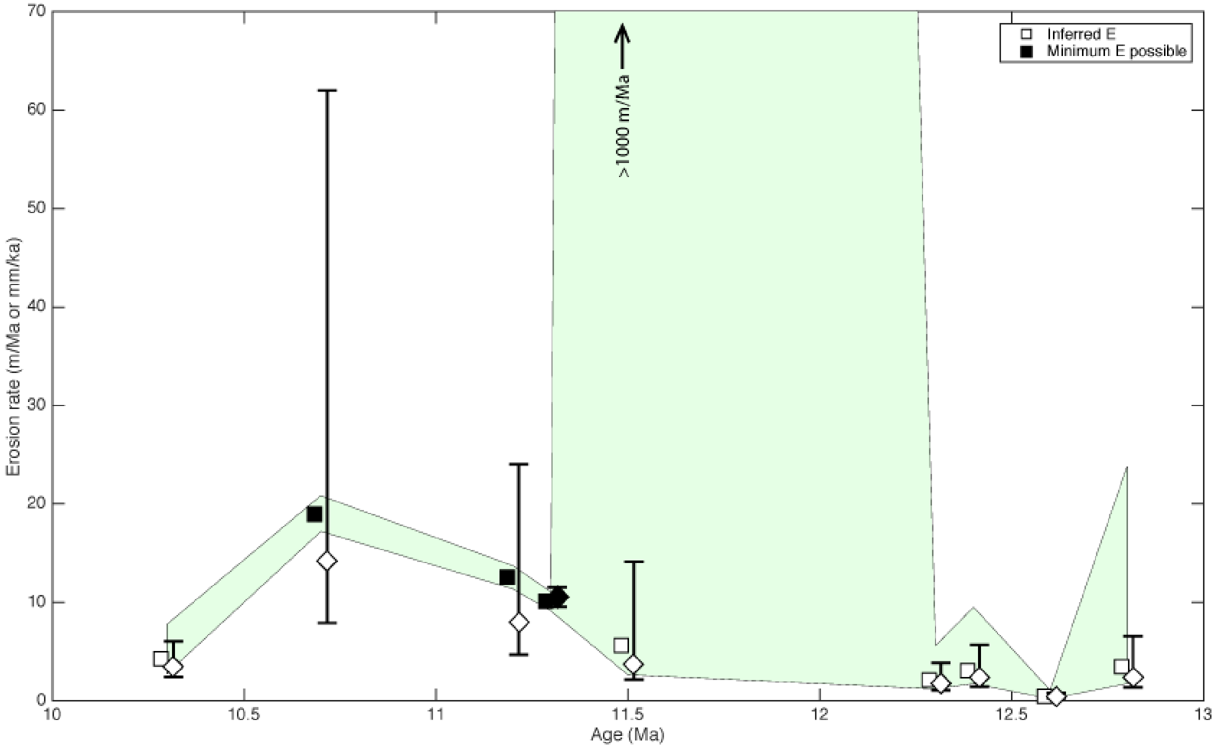

Figure S3. Paleo-denudation rates reported in the main text (diamonds with errorbars) compared to those obtained considering two 3-Ma-phases of canyon incision, where the second phase occurs at half the rate (squares within green 1σ envelope).

<u>Shielding factor during canyon incision</u>

The paleo-denudation rates reported in the main text are obtained assuming a constant shielding factor (measured in the field) during exhumation. Although the paleotopography is poorly known, the field-measured shielding factors add a good constraint on the local production rate during the recent history of canyon incision, because the relevance of $C_{incision}$ increases as the sampled material is progressively exhumed. Nevertheless, using a topographic shielding factor based on the average valley-flanks might better reproduce the average shielding throughout the entire period of canyon incision. We explore this scenario by recalculating $C_{incision}$ with a shielding factor of 0.9 (as opposed to ~0.75). The effect of this calculation is shown in Fig. S4. Although 3 of the 4 youngest samples would only yield minimum paleodenudation rates, the trend highlighted in the main text remains unaltered, if not strengthened by even higher rates at ~11 Ma.

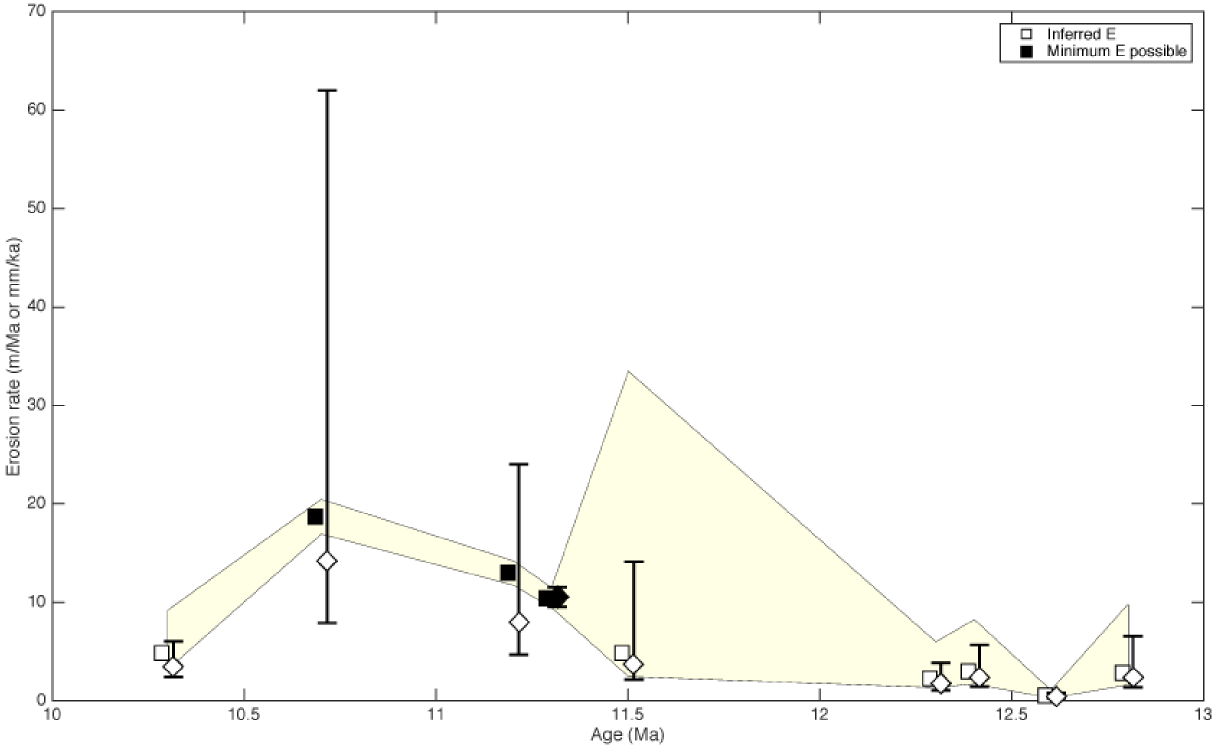

Figure S4. Paleo-denudation rates reported in the main text (diamonds with error bars) compared to those obtained using a Shielding Factor of 0.9 during canyon incision (squares within yellow 1σ envelope).

Production at depth preceding exhumation

The paleo-denudation rates reported in the main text are obtained neglecting possible production at depth between the deposition of the top of El Diablo and the start of incision. This period would have lasted approximately 3 Ma, allowing for relevant muogenic production even at several tens of meters depth. This concentration, however, becomes negligible after 6 Ma of radioactive decay, which is the approximate time of canyon incision. Fig. S5 shows that the results reported in the main text and paleo-denudation rates calculated accounting for these concentrations can be considered almost equivalent.

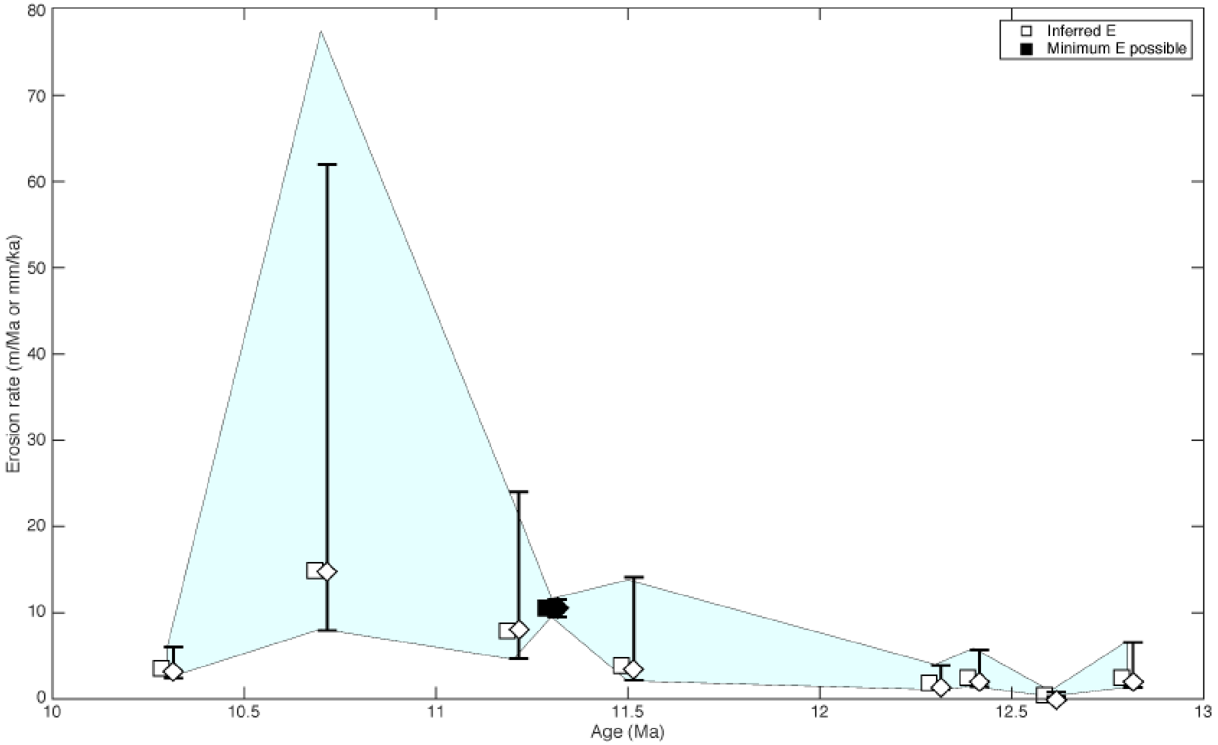

Figure S5. Paleo-denudation rates reported in the main text (white diamonds) compared to those obtained considering 3 Ma of possible production at depth before canyon incision (squares within blue 1σ envelope).

Uncertainty on the paleo-catchment production rate

The paleo-denudation rates reported in the main text are obtained neglecting additional uncertainties on the source area production rates, possibly due to fluctuations of the magnetic field strength. Such variations are not reported for times preceding ~2 Ma, but assuming that the mid-Miocene magnetic field was subject to similar fluctuations, our estimates should take into account the uncertainty deriving therefrom. In Fig. S6, the red curve shows the Lifton et al. (2014) 1-ka-Scaling Factor variations, whereas the blue curve is a 100-ka-moving average. The blue curve is more appropriate for our study, because at denudation rates <10 m/Ma (comparable to our estimates), 100 cm of eroded material would yield an apparent age >100 ka. This implies that short-term variations in production rate due to magnetic field strength need to be averaged over such integration time. Here, we therefore explore two conservative scenarios, in which the source area production rate is either increased or decreased by 11%, as suggested by the standard deviation of the blue curve in Fig. S6. The results of this sensitivity test are shown in Fig. S7, where it appears that the paleo-denudation rates would substantially remain unchanged.

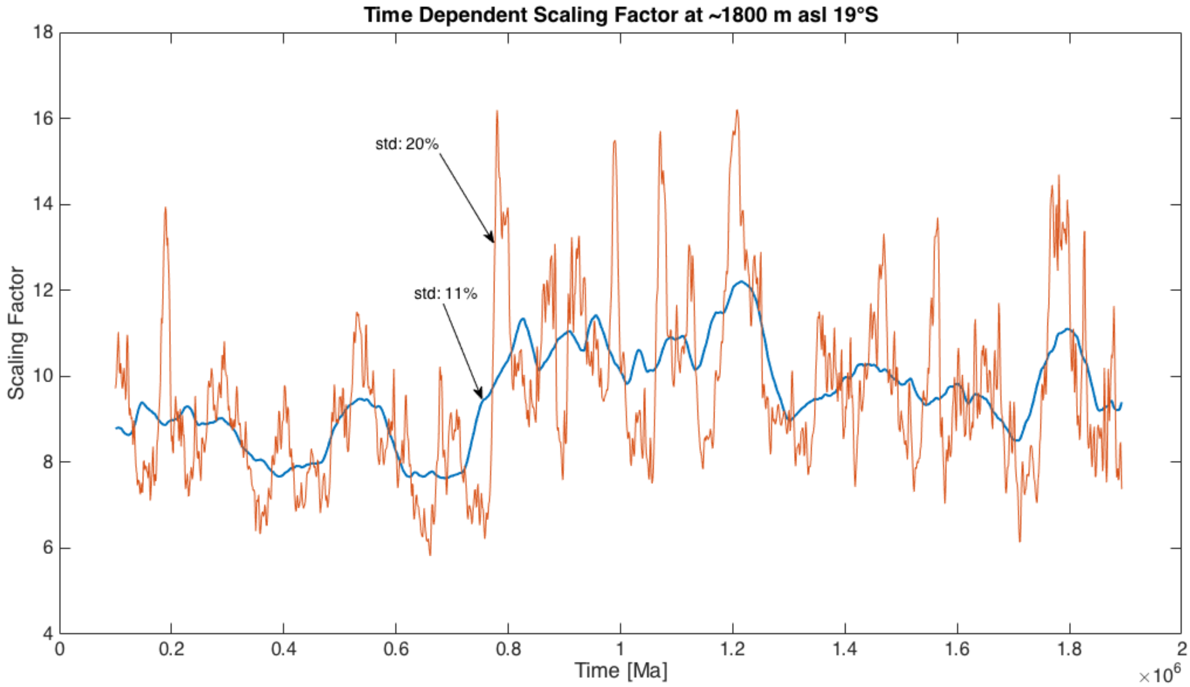

Figure S6. Red curve: Lifton et al.'s (2014) time dependent scaling factors for an elevation of 1800 m asl and a latitude of 19° S. Blue curve: 100-ka-moving average of the red curve. Std: standard deviation.

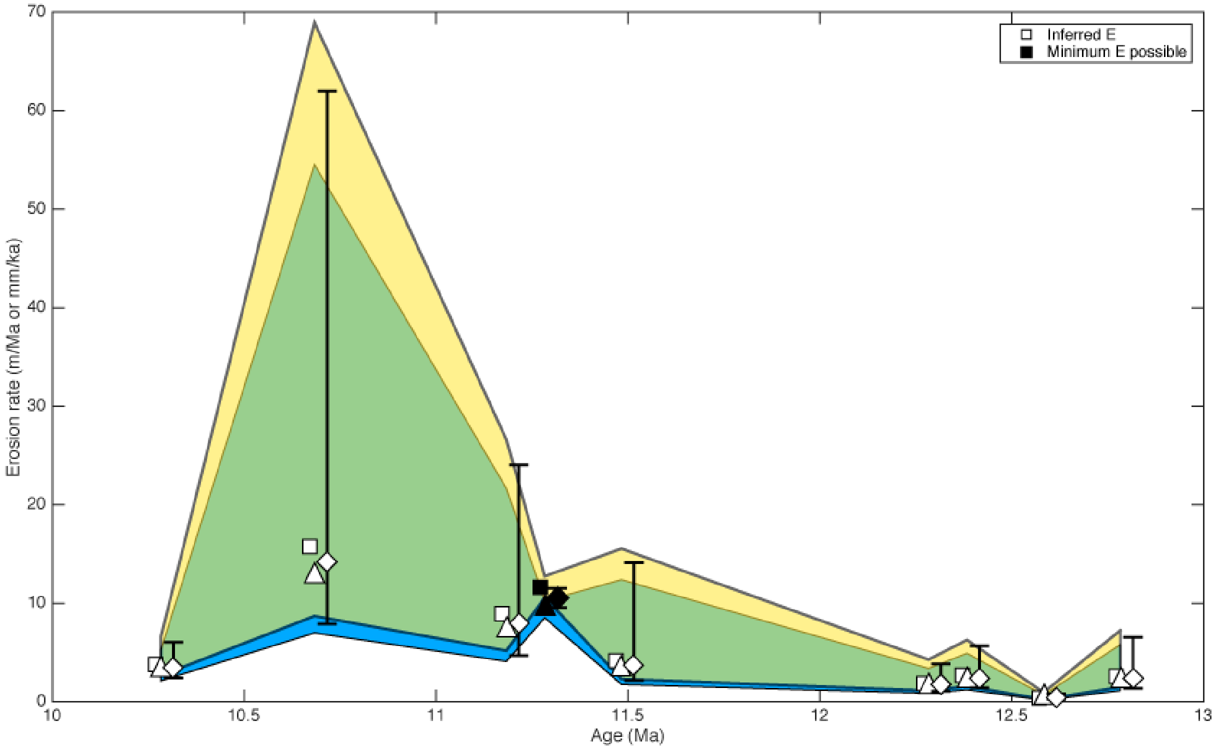

Figure S7. Paleo-denudation rates reported in the main text (diamonds with error bars) compared to those obtained increasing the source area production rate by 11% (squares in yellow envelope), and those obtained decreasing the source area production rate by 11% (triangles in blue envelope).

Magnetostratigraphic ages

The chronology of the Francia section was initially established by von Rotz et al. (2005) and later only re-assessed by Schlunegger et al. (2017), who roughly bracket it between 13-10 Ma based on new geochronological evidence (Jordan et al., 2014). Starting from the work of the latter authors, we refine the

age-correlation for each sampled layer, using the Python-based software Cupydon (Lallier et al., 2013). This tool allows an objective evaluation of the possible correlations between the reference GPTS chart (Gradstein et al., 2012) and the measured magnetic polarity zone thicknesses (von Rotz et al., 2005).

In particular each sample has been assigned the minimum and maximum age of the related polarity interval. In cases where more samples belong to the same magnetopolarity zone, the time span of the interval has been divided for the number of samples involved. Figure S8 shows this correlation and the inferred accumulation rates.

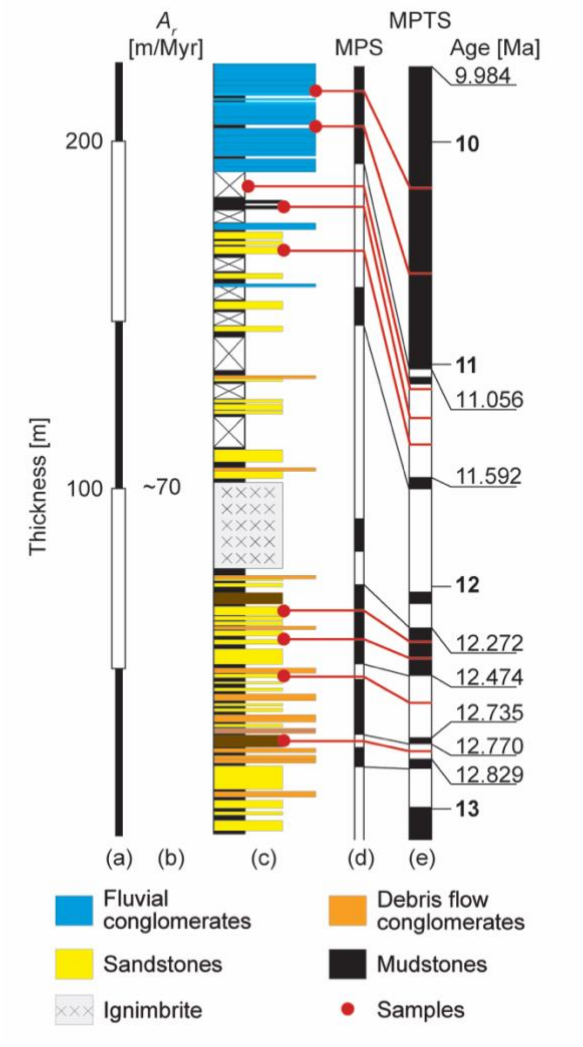

Figure S8. Information relevant for the age and burial rate of each sampled unit within the Francia section, modified after Schlunegger et al. (2017). (a) Thickness of the section, (b) inferred average burial rate, (c) stratigraphic column and sampling locations along the section (d) measured magnetostratigraphy (MPS, von Rotz et al., 2005), (e) magnetopolarity time scale (MPTS; Gradstein et al., 2012).

Compilation of present-day denudation rates

Table S1 displays the compiled and recalculated ^{10}Be-inferred current erosion rates for the western central Andes (Abbühl et al., 2011; Kober et al., 2007; Kober et al., 2009; Reber et al., 2017).

	Sample	Latitude	Longitude	Mean catchm. elev. [m a.s.l.]	^{10}Be Concentration [10^4 at/g]			Erosion rate [m/Ma]		
INCISED LANDSCAPES										
R	PRCME-1	-18.12	-70.33	2733	101.90	±	2.20	12.3	±	1.2
R	PRCME-3	-17.82	-70.50	3105	55.60	±	1.40	27.0	±	2.5
R	PRCME-401	-17.91	-70.96	2668	91.00	±	1.80	13.5	±	1.3
R	PRCME-5	-17.29	-70.99	3398	83.40	±	1.90	21.4	±	2.0
R	PRCME-6	-17.03	-71.69	3568	21.90	±	0.80	80.3	±	7.6
R	PRCME-7	-16.58	-72.73	3635	15.70	±	0.40	112.8	±	10.3
R	PRCME-8	-16.72	-72.42	3285	50.80	±	2.00	32.3	±	3.1
R	PRCME-9	-16.42	-73.12	3745	8.50	±	0.40	209.3	±	20.7
R	PRCME-12	-15.67	-74.52	2797	59.60	±	1.20	21.3	±	1.9
R	PRCME-13	-15.63	-74.64	2895	21.30	±	0.80	58.9	±	5.6
R	PRCME-1401	-14.65	-75.24	2716	27.30	±	0.80	42.3	±	3.9
R	PRCME-15	-14.34	-75.69	2204	36.60	±	1.00	24.2	±	2.2
R	PRCME-17	-13.47	-76.14	3197	18.10	±	0.50	76.7	±	7.0
R	PRCME-18	-13.32	-76.24	1967	35.40	±	0.80	21.6	±	1.9
R	PRCME-19	-13.12	-76.39	3648	28.80	±	1.10	60.7	±	5.8
R	PRCME-20	-12.67	-76.65	3294	26.10	±	0.80	56.8	±	5.3
R	PRCME-21	-12.50	-76.74	1772	26.20	±	0.80	25.4	±	2.3
R	PRCME-22	-12.25	-76.89	2568	30.60	±	0.60	34.1	±	3.0
R	PRCME-23	-11.61	-77.24	2697	14.10	±	0.50	74.6	±	6.9
R	PRCME-24	-11.07	-77.59	3134	11.30	±	0.40	112.4	±	10.4
R	PRCME-25	-10.84	-77.70	2365	25.40	±	0.60	36.0	±	3.2
R	PRCME-26	-10.66	-77.83	2407	31.70	±	0.90	29.9	±	2.7
R	PRCME-27	-8.97	-78.62	3262	22.60	±	0.90	62.2	±	6.0
R	PRCME-28	-8.42	-78.78	2246	62.00	±	2.60	14.3	±	1.4
R	PRCME-29	-8.14	-79.01	2307	42.70	±	1.40	21.2	±	2.0
R	PRCME-30	-7.32	-79.48	2292	29.90	±	0.90	29.3	±	2.7
R	PRCME-31	-6.98	-79.63	1142	35.40	±	1.00	12.6	±	1.1
R	PRCME-32	-6.45	-79.86	1423	26.20	±	0.60	20.2	±	1.8
R	PRCME-33	-6.79	-79.60	1844	22.40	±	0.70	30.2	±	2.7
R	PRCME-34	-7.82	-79.17	1985	14.40	±	0.40	49.4	±	4.3
R	PRCME-35	-9.26	-78.43	2194	16.70	±	0.40	48.3	±	4.2
R	PRCME-36	-9.48	-78.29	1938	21.90	±	0.70	32.5	±	2.9
R	PRCME-3601	-9.49	-78.27	2595	18.20	±	0.60	55.0	±	5.0
R	PRCME-37	-9.94	-78.22	1697	20.80	±	0.80	29.8	±	2.8
R	PRCME-38	-10.08	-78.15	2337	52.80	±	1.10	17.5	±	1.6
R	PRCME-39	-11.79	-76.99	2942	17.00	±	0.60	70.8	±	6.6
R	PAT-ME	-10.72	-77.77	3378	6.40	±	0.30	213.1	±	20.8
R	LIM-ME	-11.92	-76.62	3936	10.50	±	1.30	172.5	±	26.4
R	AZCE	-18.58	-69.95	3306	80.30	±	1.50	21.5	±	2.0
R	FRCE	-19.41	-69.62	3801	61.30	±	1.50	35.5	±	3.3
A	Piu6	-5.10	-80.16	1418	7.92	±	0.39	60.4	±	5.7
A	Piu8	-5.09	-80.13	1461	5.91	±	0.53	81.2	±	9.8
A	2_1	-5.04	-80.07	468	6.21	±	0.49	44.6	±	4.9

Sample		Latitude	Longitude	Mean catchm. elev. [m a.s.l.]	^{10}Be Concentration [10^4 at/g]			Erosion rate [m/Ma]		
A	Piu9	-5.02	-80.05	1708	6.24	±	0.52	88.4	±	10.3
A	2_2	-5.02	-80.05	1042	4.77	±	0.49	77.5	±	10.0
A	2_3	-4.99	-80.02	1172	3.46	±	0.48	108.1	±	17.4
A	2_4	-4.98	-80.01	1319	7.34	±	0.71	59.3	±	7.5
A	Piu10	-4.95	-80.00	2054	7.32	±	0.56	91.5	±	10.3
A	Piu13	-4.93	-79.95	2355	7.50	±	1.50	101.8	±	22.9
A	Piu12	-4.93	-79.95	2147	5.84	±	0.39	119.3	±	12.6
A	Pis1	-13.65	-75.73	3767	26.40	±	1.80	67.7	±	7.6
A	Pis1.1	-13.65	-75.73	3767	19.40	±	1.60	90.0	±	10.9
A	Pis11	-13.73	-75.89	3465	17.10	±	1.20	87.8	±	9.8
A	Pis4	-13.59	-75.36	4116	38.30	±	2.30	56.7	±	6.1
A	Pis9	-13.57	-75.54	4082	12.40	±	1.90	156.5	±	28.2
K9	LL1	-18.33	-69.87	4063	106.90	±	5.60	23.6	±	2.5
K9	LL2	-18.40	-70.02	3758	97.50	±	4.00	22.4	±	2.2
K9	LL3	-18.38	-70.28	3487	86.60	±	5.40	22.0	±	2.4
A	Pis5$^+$	-13.64	-75.74	1100	45.90	±	2.30	9.1	±	0.9
R	PRCME-11$^+$	-15.85	-74.26	2316	65.90	±	1.60	14.8	±	1.4
R	PRCME-10$^+$	-16.23	-73.62	2155	93.60	±	1.90	9.6	±	0.9
				PALEOSURFACES						
K7	1c	-18.47	-69.90	1930	1330.00	±	26.70	0.3	±	0.3
K7	5	-18.70	-69.62	3260	3080.00	±	96.00	0.3	±	0.3
K7	8b	-18.42	-69.85	1670	733.00	±	28.60	0.6	±	0.4
K7	16	-18.35	-69.62	3270	1850.00	±	62.90	0.6	±	0.4
K7	23	-18.38	-69.67	3280	3710.00	±	55.60	0.2	±	0.2
K7	36	-19.55	-70.08	1150	539.00	±	24.60	0.6	±	0.4
K7	104A	-18.37	-69.67	3235	2200.00	±	66.70	0.5	±	0.4
K7	111F	-18.23	-69.82	3435	2730.00	±	93.20	0.4	±	0.3
K7	112	-18.08	-69.68	3920	675.00	±	31.10	3.4	±	0.4
K7	201	-18.37	-69.83	3440	4160.00	±	125.00	0.2	±	0.2
K7	203	-19.20	-70.25	500	593.00	±	18.20	0.2	±	0.2
K7	301	-18.73	-69.68	2185	1470.00	±	44.60	0.3	±	0.3
K7	302	-18.73	-69.65	2590	1660.00	±	50.40	0.4	±	0.3
K7	303	-18.72	-69.65	3070	2330.00	±	70.40	0.4	±	0.3
K7	305	-18.18	-69.72	3670	1080.00	±	39.40	1.7	±	0.9
K7	310	-18.35	-69.62	3220	466.00	±	18.20	3.4	±	0.4
				WESTERN MARGIN OF THE ALTIPLANO						
A	Piu11	-4.92	-79.89	3084	223.00	±	5.40	6.3	±	0.6
A	Pis2	-13.57	-75.25	4227	322.00	±	11.00	8.2	±	0.8
K7	26	-20.30	-68.92	4205	1890.00	±	56.70	1.3	±	0.7
K9	LL4	-17.98	-69.62	4323	243.50	±	30.60	11.9	±	1.9
K9	LL5	-17.98	-69.62	4345	237.30	±	14.90	12.4	±	1.4
K7	309*	-18.23	-69.15	4510	213.00	±	11.40	14.8	±	1.6
K7	113*	-18.17	-69.50	4560	119.00	±	5.60	26.3	±	2.7

+ Samples from streams sourced in the Coastal Cordillera, not included in the compilation.

* Bedrock samples from the Lauca-Perez ignimbrite, not included in the compilation.

A: data from Abbühl et al. (2011)

K7: data from Kober et al. (2007)

K9: data from Kober et al. (2009)

R: data from Reber et al. (2017).

Table S1. Compiled topographic and cosmogenic nuclide data and relative recalculated current erosion rates.

References (supplementary)

Abbühl, L.M., Norton, K.P., Jansen, J.D., Schlunegger, F., Aldahan, A., Possnert, G., 2011. Erosion rates and mechanisms of knickzone retreat inferred from 10Be measured across strong climate gradients on the northern and central Andes Western Escarpment. Earth Surf. Process. Landforms 36, 1464–1473.

Balco, G., Stone, J.O., Lifton, N.A., Dunai, T.J., 2008. A complete and easily accessible means of calculating surface exposure ages or erosion rates from 10Be and 26Al measurements. Quat. Geochronol. 3, 174–195.

Cande, S.C., Kent, D. V., 1995. Revised calibration of the geomagnetic polarity timescale for the Late Cretaceous and Cenozoic. J. Geophys. Res. Solid Earth 100, 6093–6095.

Gradstein, F.M., Ogg, J.G., Schmitz, M.D., and Ogg, G.M., 2012, The Geologic Time Scale 2012 Volume 2: Elsevier, 438-1144 p.

Jordan, T.E., Kirk-Lawlor, N.E., Nicolás Blanco, P., Rech, J.A., Cosentino, N.J., 2014. Landscape modification in response to repeated onset of hyperarid paleoclimate states since 14 Ma, Atacama Desert, Chile. Bull. Geol. Soc. Am. 126, 1016–1046. doi:10.1130/B30978.1

Kober, F., Ivy-Ochs, S., Schlunegger, F., Baur, H., Kubik, P.W., Wieler, R., 2007. Denudation rates and a topography-driven rainfall threshold in northern Chile: Multiple cosmogenic nuclide data and sediment yield budgets. Geomorphology 83, 97–120.

Kober, F., Ivy-Ochs, S., Zeilinger, G., Schlunegger, F., Kubik, P.W., Baur, H., Wieler, R., 2009. Complex multiple cosmogenic nuclide concentration and histories in the arid Rio Lluta catchment, northern Chile. Earth Surf. Process. Landforms 34, 398–412.

Lallier, F., Antoine, C., Charreau, J., Caumon, G., and Ruiu, J., 2013, Management of ambiguities in magnetostratigraphic correlation: Earth and Planetary Science Letters, v. 371–372, p. 26–36.

Lifton, N., Sato, T., and Dunai, T.J., 2014, Scaling in situ cosmogenic nuclide production rates using analytical approximations to atmospheric cosmic-ray fluxes: Earth and Planetary Science Letters, v. 386, p. 149–160.

Reber, R., Delunel, R., Schlunegger, F., Litty, C., Madella, A., Akçar, N., Christl, M., 2017. Environmental controls on 10 Be-based catchment-averaged denudation rates along the western margin of the Peruvian Andes. Terra Nova.

Schlunegger, F., Norton, K.P., Delunel, R., Ehlers, T.A., Madella, A., 2017. Late Miocene increase in precipitation in the Western Cordillera of the Andes between 18–19°S latitudes inferred from shifts in sedimentation patterns. Earth Planet. Sci. Lett. 1, 1–12.

Strecker, M.R., Alonso, R.N., Bookhagen, B., Carrapa, B., Hilley, G.E., Sobel, E.R., Trauth, M.H., 2007. Tectonics and Climate of the Southern Central Andes. Annu. Rev. Earth Planet. Sci. 35, 747–787.

von Rotz, R., Schlunegger, F., Heller, F., Villa, I., 2005. Assessing the age of relief growth in the Andes of northern Chile: Magneto-polarity chronologies from Neogene continental sections. Terra Nov. 17, 462–471.

Wörner, G., Hammerschmidt, K., Henjes-Kunst, F., Lezaun, J., Wilke, H., 2000. Geochronology (40 Ar/39Ar, K-Ar and He-exposure ages) of Cenozoic magmatic rocks from northern Chile (18-22 S): implication for magmatism and tectonic evolution of the Central Andes. Rev. Geológica Chile 27, 205–240.

Wörner, G., Uhlig, D., Kohler, I., Seyfried, H., 2002. Evolution of the West Andean Escarpment at 18°S (N. Chile) during the last 25 Ma: Uplift, erosion and collapse through time. Tectonophysics 345, 183–198.
